# Replication of Genome Wide Association Studies on Hepatocellular Carcinoma Susceptibility Loci in a Chinese Population

**DOI:** 10.1371/journal.pone.0077315

**Published:** 2013-10-28

**Authors:** Kangmei Chen, Weimei Shi, Zhenhui Xin, Huifen Wang, Xilin Zhu, Xiaopan Wu, Zhuo Li, Hui Li, Ying Liu

**Affiliations:** 1 National Laboratory of Medical Molecular Biology, Institute of Basic Medical Sciences, Chinese Academy of Medical Sciences, School of Basic Medicine, Peking Union Medical College, Beijing, P. R. China; 2 Liver Failure Treatment and Research Center, The 302 Hospital of the People’s Liberation Army, Beijing, P. R. China; 3 Department of Infectious Disease, Affiliated Youan Hospital, Capital University of Medical Science, Beijing, P. R. China; 4 Department of Epidemiology, Institute of Basic Medical Sciences, Chinese Academy of Medical Sciences, School of Basic Medicine, Peking Union Medical College, Beijing, P. R. China; MOE Key Laboratory of Environment and Health, School of Public Health, Tongji Medical College, Huazhong University of Science and Technology, China

## Abstract

**Background:**

Genome-wide association studies (GWAS) have identified three loci (rs17401966 in *KIF1B*, rs7574865 in *STAT4*, rs9275319 in *HLA-DQ*) as being associated with hepatitis B virus-related hepatocellular carcinoma (HBV-related HCC) in a Chinese population, two loci (rs2596542 in *MICA*, rs9275572 located between *HLA-DQA* and *HLA-DQB*) with hepatitis C virus-related HCC (HCV-related HCC) in a Japanese population. In the present study, we sought to determine whether these SNPs are predictive for HBV-related HCC development in other Chinese population as well.

**Method and Findings:**

We genotyped 4 SNPs, rs2596542, rs9275572, rs17401966, rs7574865, in 506 HBV-related HCC patients and 772 chronic hepatitis B (CHB) patients in Han Chinese by TaqMan methods. Odds ratio(OR)and 95% confidence interval (CI) were calculated by logistic regression. In our case-control study, significant association between rs9275572 and HCC were observed (*P* = 0.02, OR = 0.73, 95% CI = 0.56–0.95). In the further haplotype analysis between rs2596542 at 6p21.33 and rs9275572 at 6p21.3, G-A showed a protective effect on HBV-related HCC occurrence (*P*<0.001, OR = 0.66, 95% CI = 0.52–0.84).

**Conclusion:**

These findings provided convincing evidence that rs9275572 significantly associated with HBV-related HCC.

## Introduction

Liver cancer is the fifth most frequently diagnosed cancer worldwide but the second most frequent cause of cancer death [1]. Hepatocellular carcinoma (HCC) accounts for between 70% and 85% of primary liver cancers, and ranks fifth and sixth as causes of cancer mortality worldwide in men and women, respectively [2]–[3]. Globally, there are more than 250,000 new cases of HCC and an estimated 500,000–600,000 deaths due to this disease annually [4]. Eastern Asia is the geographic area at highest risk of HCC, and China accounts for 55% of all HCC cases worldwide [1]. Hepatitis B virus (HBV) and hepatitis C virus (HCV) infections are the leading cause of HCC in the world [4]. In Western countries and Japan, infection with HCV is the more common cause of HCC, while in Asia and developing countries, HBV is more common [5]. Furthermore, accumulated evidences in molecular genetics indicate that single nucleotide polymorphisms (SNPs) in immune response and tumorigenesis related genes are associated with susceptibility to HCC [6]–[9]. Recently, a number of Genome-wide association studies (GWAS) have identified several new loci associated with the risk of HCC, such as SNPs in the gene *KIF1B*, *MICA*, *HLA-DQA/DQB*, *STAT4* and *HLA-DQ*, respectively [10]–[12].

Two independent GWAS have been performed to identify novel susceptibility loci associate with HBV-related HCC [10], [12]. Among these studies, Zhang et al. found one susceptibility locus (rs17401966) in *KIF1B* at chromosome 1p36.22 [10], Jiang et al. confirmed two other loci, rs7574865 in the *STAT4* at 2q32.2–2q32.3 and rs9275319 in *HLA-DQ* at 6p21.3 [12]. Moreover, a GWAS of Japanese population conducted by Kumar et al. identified two susceptibility loci for HCV-related HCC, with lead SNPs rs2596542 located 4.7 kb upstream of *MICA* on 6p21.33 and rs9275572 located between *HLA-DQA* and *HLA-DQB* on 6p21.32 [11]. Although the mechanism of chronic HBV and HCV infection is not identical, they share some common characteristics to induce HCC [13]. So, we speculated these two SNPs associated with HCV-related HCC patients would be also associated with HBV-related HCC patients.

Given the confirmatory results from several studies in multiple populations, the above hypothsis and the design of TaqMan probe, we focus on four polymorphisms, *KIF1B* rs17401966, *STAT4* rs7574865, *MICA* rs2596542 and *HLA-DQA/HLA-DQB* rs9275572, to replicate in a HBV-related HCC case-control study among Chinese Han population.

## Materials and Methods

### Subjects

The subjects enrolled in the present study consisted of 506 HBV-related HCC cases and 772 CHB controls. All subjects were recruited from Beijing Youan Hospital (Beijing) and the 302 Hospital of the People’s Liberation Army (Beijing) from Oct 2005 to Jul 2010.

Subjects with CHB were identified with the following diagnostic criteria: liver ultrasonography confirmed; serum levels of alanine aminotransferase (ALT) and aspartate aminotransferase (AST) continuously >40 IU/L; HBsAg seropositive for at least 6 months and serum HBV DNA >2000 copies/ml. The diagnosis of HCC was identified based on clinical evidence obtained from liver function tests, serum immunologic marker screening, pathologically confirmed, liver ultrasonography(US)/computed tomography(CT) and proved not to have other cancers.

Subjects were considered smokers if they smoked up to 6 months before the date of cancer diagnosis for HCC cases or the date of interview for CHB controls. An alcohol drinker was defined as someone who consumed alcohol at least once per week for at least 6 months.

The subjects were excluded if: (1) there was evidence of past or current infection with other hepatitis viruses or hepatitis not caused by HBV; (2) they were not of Han ethnicity. The study was carried out in accordance with the guidelines of the Helsinki Declaration after obtaining written informed consent from all the subjects and was approved by the ethics committee of the Institute of Basic Medical Sciences, Chinese Academy of Medical Sciences.

### SNP Selection and Genotyping

Genomic DNA was extracted from peripheral blood by using a salting-out protocol [14]. TaqMan assays for four SNPs were purchased from Applied Biosystems (Foster City, CA) and run according to the manufacturer. Briefly, each 10 µL TaqMan reaction contain 40 ng of genomic DNA, primers, probes, and 2×GoldStar TaqMan Mixture (CWBIO, Beijing, China) and was performed with the following procedure: 95°C for 10 min, followed by 40 cycles of 92°C for 15 s, and 64°C for 1 min in the real-time PCR instrument. Primers and TaqMan probes used are listed in Table S1. All the samples were successfully genotyped. To ensure quality control, 5% samples were randomly selected and directly sequenced, and we obtained 100% identical results.

### Statistical Analysis

We used 2×2 and 2×3 contingency tables for comparing allele and genotype frequencies between subjects with HCC and CHB. Multiple logistic regression models (dominant, recessive, and log-additive) were used for calculating the Odds ratio (OR), 95% confidence interval (CI), and corresponding *P* value, with adjustment for sex and age. Nonsuperiority test was conducted to confirm the absence of association between SNPs and risk of HCC [15]. Multiple comparison adjustment based on the false discovery rate (FDR) principle was used to avoid the false positive results [16]. All statistical analyses were performed using the Statistical Package for the Social Sciences (SPSS; version 12.0), the software program SNPStats, and the Statistical Analysis System Software (version 9.2; SAS Institute, Cary NC) [17]. We estimated linkage disequilibrium (LD) values (D′), r^2^ values, and haplotypes by using the SHEsis online software [18]. A *P* value of <0.05 was used as the criterion for statistical significance.

## Results

The demographic details of the patients and control subjects enrolled in the study are shown in Table 1. The significant differences found between cases and controls for age, gender were controlled for in the multivariate analysis.

**Table 1:** Clinical features of the subjects included in the study.

	HCC	CHB	*P*
Number	506	772	
Age, years, mean ±SD	53.9±10.6	35.7±11.8	<0.001^a^
Gender (male/female)	425/81	572/199	<0.001^b^
Smoking (Yes/No)	246/260	191/499	<0.001^b^
Drinking (Yes/No)	261/245	217/470	<0.001^b^

a Mann-Whitney U test.

b Chi-square test.

The genotype distributions and allelic frequencies of 4 SNPs were represented in Table 2. As shown in the Table, rs9275572 was significantly associated with HCC. The frequency of the A allele was 20.2% in HBV-related HCC patients vs. 24.4% in CHB patients (*P* = 0.01, OR = 0.78, 95% CI = 0.64–0.95). The frequencies of the rs9275572 genotypes also differed significantly between the two groups (*P = *0.02). This association remains significant after adjusting for age and gender under additive model (*P* = 0.02, OR = 0.73, 95% CI = 0.56–0.95), dominant model (*P* = 0.02, OR = 0.71, 95% CI = 0.53–0.96) and recessive model (*P* = 0.01, OR = 0.41, 95% CI = 0.20–0.83). The remaining 3 SNP loci, rs2596542, rs17401966 and rs7574865, did not significantly differ between the HCC patients and CHB patients.

**Table 2:** Associations between GWAS-identified SNPs and HBV-related HCC in a Chinese population.

			Allele, n (%)				Genotype, n (%)			Additive model#		Dominant model#		Recessive model#	
	1/2	1	2	OR (95% CI)	*P*	11	12	22	*P*	OR (95% CI)	*P*	OR (95% CI)	*P*	OR (95% CI)	*P*
rs2596542	A/G														
HCC (n = 506)		284(28.1%)	728(71.9%)	1.11(0.93–1.33)	0.24	42(8.3%)	200(39.5%)	264(52.2%)	0.51	0.88(0.69–1.13)	0.32	1.15(0.86–1.53)	0.35	1.15(0.67–1.98)	0.61
CHB (n = 772)		401(26.0%)	1143(74.0%)			54(7.0%)	293(38.0%)	425(55.1%)							
rs9275572	A/G														
HCC (n = 506)		204(20.2%)	808(79.8%)	0.78(0.64–0.95)	0.01	17(3.4%)	170(33.6%)	319(63.0%)	0.02	0.73(0.56–0.95)	0.02	0.71(0.53–0.96)	0.02	0.41(0.20–0.83)	0.01
CHB (n = 772)		377(24.4%)	1167(75.6%)			50(6.5%)	277(35.9%)	445(57.6%)							
rs17401966	C/T														
HCC (n = 503)		320(31.8%)	686(68.2%)	1.17(0.99–1.40)	0.07	63(12.5%)	194(38.6%)	246(48.9%)	0.06	0.96(0.77–1.20)	0.72	1.01(0.76–1.34)	0.95	1.32(0.83–2.09)	0.24
CHB (n = 772)		439(28.4%)	1105(71.6%)			65(8.4%)	309(40.0%)	398(51.6%)							
Rs7574865	A/C														
HCC (n = 501)		287(28.6%)	715(71.4%)	0.90(0.75–1.07)	0.23	35(7.0%)	217(43.3%)	249(49.7%)	0.24	0.87(0.68–1.10)	0.24	0.92(0.69–1.23)	0.57	0.70(0.42–1.18)	0.18
CHB (n = 772)		477(30.9%)	1067(69.1%)			75(9.7%)	327(42.4%)	370(47.9%)							

# *P* values and OR were adjusted for age and gender in each additive, dominant or recessive genetic model, respectively.

The LD analysis of 2 SNPs (rs2596542, rs9275572) at chromosome 6p21.3 was performed, and showed no apparent LD (D′≤0.179, r^2^≤0.026). A haplotype analysis between these two SNPs was further conducted, and 4 haplotypes were observed (Table 3). Out of which, the frequency of the G-A haplotype was significantly higher in the CHB controls than in the HBV-related HCC patients (15.5% vs. 10.8%, P = 0.0007). The OR for the G-A haplotype was 0.66, which is stronger than that of rs9275572 alone (OR = 0.73), suggesting that allele G of rs2596542 may strengthen the protective impact of allele A of rs9275572 in HBV-related HCC.

**Table 3:** The results of haplotype analysis of rs2596542 and rs9275572.

Haplotype	HCC (%)	CHB (%)	*P*	OR (95%CI)
	(2n = 1012)	(2n = 1544)		
A-A	95(9.4%)	138(9.0%)	0.72	1.05(0.80–1.38)
A-G	189(18.7%)	263(17.0%)	0.28	1.12(0.91–1.38)
G-A	109(10.8%)	239(15.5%)	0.0007	0.66(0.52–0.84)
G-G	619(61.2%)	904(58.6%)	0.19	1.11(0.95–1.31)

To avoid the false positive results for rs9275572, we have conducted an adjustment for multiple testing based on the false discovery rate (FDR) principle. After correction, the frequency for allele (*P* = 0.04) and the association under recessive model (*P* = 0.04) remained significant at the 5% level, the other effects were no significant (*P* = 0.08 for genotype, *P* = 0.08 under additive model, *P* = 0.08 under dominant model).

Nonsuperiority test was used to confirm the absence of association between rs25969542, rs17401966 and rs7574865 with HBV-related HCC, respectively. The null hypothesis is that the frequency of rs25969542*A, rs17401966*C and rs7574865*A in HCC patients is greater by Δ compared to the frequency in CHB controls. The Δ was set (5% for rs17401966; 3% for rs7574865) based on the lowest difference of minor allele between HCC and CHB patients in Chinese population in the previous study. There was no reference data for rs2596542 significantly associated with HCC in Chinese population in recent study. The corresponding nonsuperiority *P*-values for rs17401966 and rs7574865 was 0.0001 and 0.3425, respectively, which support the absence of association between rs17401966 and HBV-related HCC.

## Discussion

In this study, we attempted to replicate, in a Chinese population, the associations between the 4 SNP loci and the risk of HCC, which were identified in previous GWAS study. The result showed that, rs9275572 between *HLA-DQA* and *HLA-DQB*, was significantly associated with HBV-related HCC risk, although it was identified by Kumar et al. to be associated with HCV-related HCC in Japanese patients. However, there was no association between the other 3 SNPs and HCC risk.

The GWAS study by Kumar et al. identified two susceptibility loci for HCV-related HCC, with lead SNPs rs2596542 and rs9275572. Importantly, the frequency of the minor allele A of rs9275572 was reported to be a risk factor for HCV-related HCC [11]. Interestingly, in our study the minor allele A appeared to have a protective impact on HBV-related HCC development, which represented an inverse association as compared to the result in Japanese population. To some extent, this result seemed puzzling, but the following points should be noted.

First, chronic infection with HBV and HCV is one of the most important risk factors of the development of HCC. Although there is certain similarity in clinical manifestations of hepatitis induced by these viruses and creating background for subsequent development for HCC, their molecular organization, replication strategy and functions of constituent proteins are different. HBV and HCV are two different viruses. HBV is a DNA-containing virus, which belongs to hepadnaviruses, whereas HCV is a RNA-containing virus of the flavivirus family [13], [19]. It is also reported that HBV and HCV infections tend to suppress each other in dual virus infections. HCV super infection is seen to reduce HBsAg expression and promote its clearance [20]. Thus, on the basis of these findings, different mechanisms of liver carcinogenesis might operate in HBV related and in HCV related chronic inflammation and cirrhosis. It is possible that rs9275572*A involved in two different pathways in HBV and HCV induced HCC, respectively. This may explain our findings rs9275572*A has opposite effect on HBV and HCV related HCC development. Second, extensive allele diversity is observed in *HLA* locus associations with susceptibility regarding HBV and HCV infections and disease progression in different global ethnic populations. However, the specific *HLA* associations with HBV and HCV infections are different, agreeing to their differences in viral properties and disease pathogenesis. It had been found HLA DQB1*0301 are protective for HCV infections, but are also associated with chronic HBV infection [21]. These evidences support that rs9275572*A may have a risk impact on HCV-related HCC but a protective impact on HBV-related HCC. Third,a Swiss study of HCV-related HCC by Christian M. Lange et al. suggested a protective role for the rs2596542 A allele, which makes an inverse association compared with the Japanese study [4], [22]. In this regard, this result may support our findings about rs9275572*A, it could be possible that a locus make opposite influence in different ethnic groups due to distinct genetic backgrounds and environmental pressures. Taken together, the disparity of association may suggest the different genetic background of the susceptibilities for HCV-related HCC and HBV-related HCC.

After the GWAS with respect to these SNPs, many replication studies were with inconsistent results. The findings for rs17401966 and rs7574865 have been replicated in some HBV-related HCC studies [23]–[24]. Noteworthy, nonsuperiority test conducted in this study provide the possibility of observing a lack of association by chance between rs7574865 and HCC (*P* = 0.3425). However, the nonexistence of association between rs17401966 and HCC was confirmed by the nonsuperiority test (*P* = 0.0001). Interestingly, the meta-analysis performed by Wang et al. indicated that rs17401966*G significantly reduced the risk for HCC in large-sample-size cohorts, but did not in small-sample-size cohorts [23]. Therefore, our negative result about rs17401966 and HCC risk might be attributed to the relatively small sample sizes. Moreover, by now, no replication study between rs2596542 and HBV-related HCC had been reported, nonsuperiority test about this locus cannot be conducted, further studies are needed.

There are some limitations in this study should be acknowledged. First, the sample size of our case-control study was relatively small. Second, the non-HBV control was not involved in the present study, further study will be needed to confirm whether rs9275572 has an association with development of CHB.

In conclusion, our study provided convincing evidence of the genetic involvement of rs9275572 polymorphism in HBV-related HCC susceptibility. Further studies including larger sample sizes and different ethnic populations should be taken to investigate the mechanisms underlying the role of this SNP in both HBV-related HCC and HCV-related HCC.

## Supporting Information

Table S1 **Primers and Probes used in TaqMan Genotyping.**
(DOC)
