# Supplementary material for: Replication of Genome Wide Association Studies on Hepatocellular Carcinoma Susceptibility Loci in a Chinese Population
Source: PLoS One. 2013 Oct 28;8(10):e77315. doi: 10.1371/journal.pone.0077315 (PMC3810470; doi:10.1371/journal.pone.0077315)
Supplement: Table S1 — Primers and Probes used in TaqMan Genotyping. (DOC) [file pone.0077315.s001.doc]

**Table S1****.** Primers and Probes used in TaqMan Genotyping.

| Primers and probes | Sequence (5’ – 3’) |
| --- | --- |
| rs2596545 |  |
| Forward primer of PCR | CCCACATCTCCACTACCACA |
| Reverse primer of PCR | AAAAGTATGCTGGGCACATCT |
| FAM labeled G allele | CCCAAAGAACAGCTACACgATAAAATT |
| HEX labeled A allele | CCCAAAGAACAGCTACACaATAAAATTC |
| rs9275572 |  |
| Forward primer of PCR | CTTCAAAGATGTGGACTTTAGG |
| Reverse primer of PCR | CCTTTTAATCTTGAACTTAGACTAGG |
| FAM labeled G allele | CTGCTCCATAGCAgCTTCATTAAAG |
| HEX labeled A allele | TGCTCCATAGCAaCTTCATTAAAGG |
| rs17401966 |  |
| Forward primer of PCR | ATTTCCCTGCTTTGAAAATTTG |
| Reverse primer of PCR | TCCAGCACTTAATGAAAACACATA |
| FAM labeled G allele | TCTAAGAACACTTGACTCAATAcGGACTCA |
| HEX labeled A allele | CCTCTAAGAACACTTGACTCAATAtGGACTC |
| rs7574865 |  |
| Forward primer of PCR | AGAAGAAAGAAAAAAAATCCCCT |
| Reverse primer of PCR | GGAAAATTACATGAGTGTGTATGC |
| FAM labeled G allele | ATAACCACTATTcACATTTTGGTCACCAAC |
| HEX labeled A allele | TAACCACTATTaACATTTTGGTCACCAACTT |
